# Regulation and Functions of ROP GTPases in Plant–Microbe Interactions

**DOI:** 10.3390/cells9092016

**Published:** 2020-09-02

**Authors:** Stefan Engelhardt, Adriana Trutzenberg, Ralph Hückelhoven

**Affiliations:** Phytopathology, TUM School of Life Sciences Weihenstephan, Technical University of Munich, Emil-Ramann-Straße 2, 85354 Freising, Germany; stefan1.engelhardt@tum.de (S.E.); a.trutzenberg@tum.de (A.T.)

**Keywords:** Arabidopsis, barley, plant immunity, ROP GTPase, rice, disease susceptibility

## Abstract

Rho proteins of plants (ROPs) form a specific clade of Rho GTPases, which are involved in either plant immunity or susceptibility to diseases. They are intensively studied in grass host plants, in which ROPs are signaling hubs downstream of both cell surface immune receptor kinases and intracellular nucleotide-binding leucine-rich repeat receptors, which activate major branches of plant immune signaling. Additionally, invasive fungal pathogens may co-opt the function of ROPs for manipulation of the cytoskeleton, cell invasion and host cell developmental reprogramming, which promote pathogenic colonization. Strikingly, mammalian bacterial pathogens also initiate both effector-triggered susceptibility for cell invasion and effector-triggered immunity via Rho GTPases. In this review, we summarize central concepts of Rho signaling in disease and immunity of plants and briefly compare them to important findings in the mammalian research field. We focus on Rho activation, downstream signaling and cellular reorganization under control of Rho proteins involved in disease progression and pathogen resistance.

## 1. Introduction

**Figure 1:**
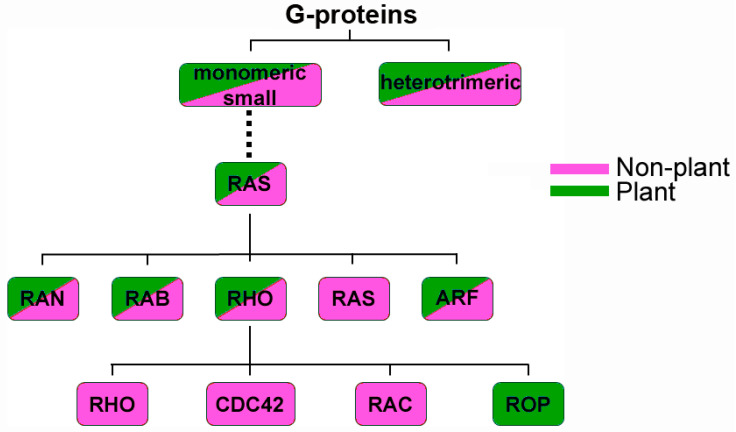
Important classes of G-proteins in eukaryotes. Heterotrimeric versus monomeric small GTPases as present in plants and non-plant eukaryotes (referring only to animals and true fungi here). Rat sarcoma (RAS) proteins are absent from plants, whereas Rho proteins of plants (ROPs) build an exclusive class of Rho family GTPases in plants.

A multitude of essential cellular processes in eukaryotic organisms such as cell polarization, vesicle trafficking and cytoskeleton organization are regulated by small GTPases or G-proteins. Due to their high guanine nucleotide binding affinity, G-proteins are molecular switches which can circle between an inactive, GDP-bound state and the active, GTP-bound conformation [1], the latter allowing direct interaction with downstream signaling executors (G-protein downstream effectors are referred to as executors here to distinguish them from pathogen virulence effectors). G-proteins can be divided into two major classes, monomeric small G-proteins and heterotrimeric G-proteins (Figure 1). Five eukaryotic members of the monomeric small G-protein family form the Ras superfamily, which includes Ras, Ran, Arf, Rab and Rho proteins [2,3]. In animals and fungi, the Rho family can be further divided into Rho, Rac and Cdc42 subfamilies. In plants, however, a unique Rho subfamily exists which shows high similarity to the animal Rac (rat sarcoma (RAS)-related C3 botulinum toxin substrate) subfamily, and its members were thus originally called plant RACs and more recently ROPs (Rho proteins of plants) [4,5,6,7,8,9]. A structure–function analysis revealed five conserved G-box motifs in the catalytic G-domain. A hypervariable region at the C-terminus undergoes post-translational lipid modifications, which give rise to two different classes of ROPs. The cysteine residue in the C-terminal CaaL motif of type I ROPs is prenylated, while cysteine residues of GC-CG boxes in the C-terminal part of type II ROPs are S-acylated. Lipid modifications, together with a proximal polybasic region that is rich in arginine and lysine residues, are required for plasma membrane association [9,10], from where activated ROPs relay signaling. Naturally, like other G-proteins, ROP activity needs to be spatiotemporally controlled in order to prevent fatal aberrant downstream signaling. The switch from the inactive to the activated state and back is regulated by different regulatory co-factors. A guanine nucleotide exchange factor (GEF) facilitates the nucleotide exchange of GDP to GTP by weakening the GDP nucleotide affinity, resulting in GDP release, GTP binding and hence ROP activation [1]. In the activated state, ROPs do not only interact with downstream signaling executors, but also with GTPase-activating proteins (GAPs), which enhance the weak intrinsic enzymatic GTPase activity of ROPs. GTP hydrolysis leads to ROP inactivation, thus GAPs are considered to be negative regulators of ROP activity [10,11]. The function of the third group of regulatory proteins, GDP dissociation inhibitors (GDIs), is based on their chaperoning properties to keep ROPs in the cytoplasm and to prevent proteasomal degradation for potential reactivation by GEFs [10,12]. ROPs regulate a multitude of cellular mechanisms which include cytoskeleton organization leading to cell size and shape development, plant hormone signaling and responses to pathogens [10]. The latter becomes increasingly important, also due to current food security discussions. The involvement of ROPs in plant–microbe interactions has been known for quite a long time, and in this review we aimed to summarize the current knowledge and compare the role of specific ROPs in immunity, disease and mutualistic symbiosis.

## 2. Rice OsRAC1 Acts in Plant Immunity

**Figure 2:**
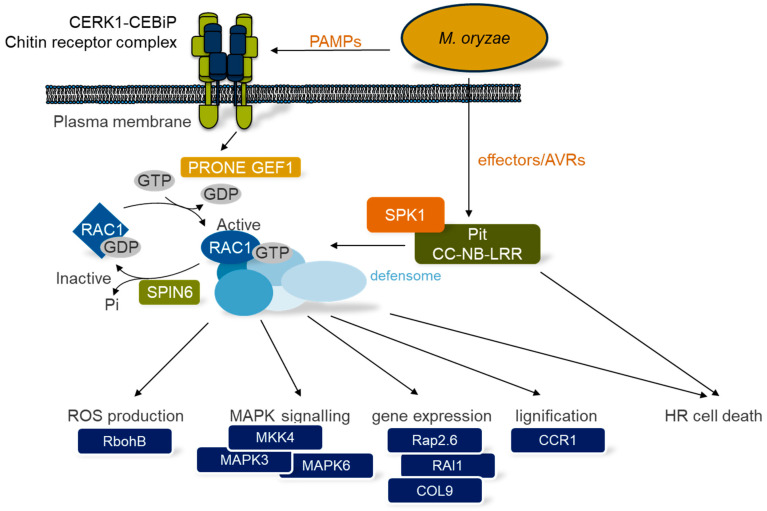
Rice MTI and ETI signaling via OsRAC1 during interaction with a parasitic fungus. Rice OsRAC1 is activated via diverse GEF proteins (PRONE GEF1 and SPK1) in either chitin-triggered cell surface PRR-mediated or effector-triggered NLR-mediated immunity.

When plants interact with microbes, they sense endogenous (phytocytokines and damage-associated molecular patterns (DAMPs)) and exogenous signals (non-self, microbe-associated molecular patterns (MAMPs) and effectors) with the help of cell surface receptors (pattern recognition receptors (PRRs), receptor-like proteins (RLPs) and receptor-like kinases (RLKs)) or intracellular nucleotide-binding oligomerization domain (NOD)-like receptors (NLRs) [13,14,15,16]. Those immunogenic signals are then transduced into characteristic immune reactions, which depending on the eliciting molecule is often called MAMP-triggered immunity (MTI) or effector-triggered immunity (ETI). Extensive studies over the last two decades have revealed the complex involvement of *Oryza sativa* RAC1 (OsRAC1) in both MTI and ETI-related responses during the rice–*Magnaporthe oryzae* interaction [17,18]. OsRAC1 is one of seven RAC/ROP members in rice [19] and belongs together with OsRAC2–4 to the type II class of RAC/ROP proteins. A tissue-specific expression analysis showed no overall redundancy suggesting that each RAC/ROP protein regulates individual signaling pathways [20]. Regarding disease resistance, OsRAC3 and OsRAC7 most likely do not play an important role, while OsRAC4 and OsRAC5 rather appear to be negative regulators of rice blast resistance. In contrast, OsRAC1 is not only the most studied of all rice RAC proteins, it has also been shown to be the key positive regulator in early rice PRR- and NLR-mediated immune signaling [21], and we will review below previous research achievements together with recent findings (Figure 2).

### 2.1. Activation of OsRAC1 in MTI and ETI

In order to mediate its function to interact with downstream signaling partners and therefore to trigger distinct cellular responses, OsRAC1 like other RAC/ROP proteins requires activation. This is achieved upon direct interaction with GEFs that facilitate the release of GDP and the binding of GTP, thereby rendering it active. In MAMP-triggered immunity, fungus-derived chitin and sphingolipids induce GEF-mediated activation of OsRAC1 [21,22,23]. A diffuse B-cell lymphoma homology-pleckstrin homology (DH-PH) domain-containing RAC/ROP GEF in rice resembling human switching B-cell complex-associated protein 70 (SWAP70) has been found to interact with OsRAC1, and the DH domain revealed GEF activity toward OsRAC1 in vitro [24]. It still remains to be seen though, if the observed OsSWAP70-mediated reactive oxygen species (ROS) production and defense gene expression in rice upon chitin treatment is indeed regulated via OsRAC1, since transgenic *OsSWAP70* RNAi rice plants are not compromised in their defense responses to rice blast infection [23]. This is in contrast to *Arabidopsis thaliana* AtSWAP70 GEF that is involved in MTI and ETI against *Pseudomonas syringae* [25]. The recognition of fungal sphingolipid elicitors might also lead to an alternative, yet to be discovered, activation mechanism of OsRAC1. In this signaling pathway, the α-subunit of a trimeric G-protein is involved, and it was reported that Gα is part of the same protein complex that contains OsRAC1 [22,26], but it remains to be seen how the GDP/GTP exchange in OsRAC1 is accomplished. 

A much more profound knowledge about upstream events of OsRAC1 activation during fungal elicitor-induced MTI emerged after the discovery of a plant-specific ROP nucleotide exchange (PRONE)-type GEF, named OsRacGEF1 [23], that directly interacts with OsRAC1 in vitro and in planta. Furthermore, the expression of pathogenesis-related genes in *OsRacGEF1* RNAi plants was significantly suppressed upon exposure to fungal-derived chitin, and these plants were also more susceptible to fungal infection compared to wild-type plants. OsRacGEF1 is chitin-dependently activated by the PRR receptor-like kinase module OsCEBiP/OsCERK1 via phosphorylation of a specific serine residue [23,27,28]. It has recently been reported that OsRacGEF1 can form homodimers and heterodimers with OsRacGEF2 at the plasma membrane (PM) and the endoplasmic reticulum (ER), but only OsRacGEF1 binds directly to OsCERK1 at the ER [29]. This RLK-GEF complex is then transported to the PM via a Sar1-dependent vesicular pathway to associate with OsRAC1, eventually forming a stable immune complex [30]. It is tempting to speculate that the fine-tuning of MTI responses in rice regulated by OsRAC1 is achieved by the constitution of the OsCERK1-GEF homo/heterodimer complex. In any case, currently available data strongly point toward OsRacGEF1 being a primary GEF for OsRAC1 activation in rice MTI.

The activation of OsRAC1 in NLR-mediated immune signaling in rice is achieved via a different mechanism. Defense responses mediated by the NLR genes *Pia* and *Pit* require OsRAC1; GEF activity, however, could not be found in these resistance proteins [20,31,32]. Recently, it was demonstrated in vitro and *in vivo* that *Oryza sativa* SPIKE1 (OsSPK1), a dedicator of cytokinesis (DOCK) family GEF, is involved in rice blast resistance signaling via Pia and Pit, most likely by facilitating OsRAC1 activation [33]. OsRAC1 forms a complex with Pit at the plasma membrane by directly interacting with the NB-ARC (nucleotide-binding adaptor shared by APAF-1, R-proteins and CED-4) domain of the NLR protein [31]. For OsSPK1 activation, the recruitment of OsSPK1 from the cytosol and endomembrane to the plasma membrane is required [33]. Additionally, the essential Pit localization at the plasma membrane is ensured by palmitoylation in its N-terminal coiled-coil (CC) region [34]. OsSPK1 binds the CC domain of Pit, and the formation of this putative ternary complex is believed to be crucial for OsRAC1 activation [33]. The OsSPK1-OsRAC1 module is probably also involved in Pia-mediated disease resistance to rice blast fungus, since OsSPK1 interacts directly with RGA4 [33], one partner of the Pia co-immune receptor pair [35,36]. It will be interesting to see if OsRAC1 is similarly activated by OsSPK1 upon RGA5-mediated recognition of *Magnaporthe oryzae* effector Avr-Pia, thereby releasing constitutively activated RGA4 to either form an RGA4-OsSPK1-OsRAC1 supercomplex or to interact with further downstream signaling partners leading to programmed cell death [37].

### 2.2. Interactors of Activated OsRAC1

Rice MTI responses regulated by OsRAC1 require a bunch of proteins besides the abovementioned OsCEBiP/OsCERK1-OsRacGEF1-OsRAC1 module. OsRAC1 is but one part of a massive complex named ‘defensome’ [18] containing at least 15 components (Figure 1). Some of them, like the chaperone complex Hop/Sti1-HSP90 (Hsp70-Hsp90 organizing protein/stress inducible protein 1-heat shock protein 90), ensure the efficient transport of OsCERK1 from the ER to the plasma membrane [30]. RAR1 (required for Mla12 resistance 1), SGT1 (suppressor of the G_2_ allele of *skp1*), and Hsp90 and Hsp70 (heat shock proteins 90 and 70) are also integral defensome members and their implications in rice innate immunity have been demonstrated [30,38]. However, these chaperones and co-chaperones have stabilizing or scaffolding functions supporting the integrity of the defensome, and direct interactions with OsRAC1 have not yet been reported. OsRAC1 has instead been demonstrated to interact directly with the N-terminal region including the two EF-hand motifs of NADPH oxidase OsRbohB (respiratory burst oxidase homolog B) [39,40,41]. This interaction, which supports NADPH oxidase enzyme activity, nicely demonstrates the regulatory role that OsRAC1 plays in the production of ROS. More importantly, for chitin-induced immune reactions via ROS signaling, the OsRAC1-OsRbohB module is required to be localized in distinct membrane microdomains that are characterized by specific levels of 2-hydroxy fatty acid-containing sphingolipids [42]. Two genes encoding fatty acid 2-hydroxylases were shown to be essential for fungal chitin-induced ROS production enabling the necessary dynamics of OsRAC1 and OsRbohB. Another direct interactor of activated OsRAC1 is OsCCR1, a cinnamoyl-CoA reductase, that is involved in lignin biosynthesis [43]. Lignin polymerization requires H_2_O_2_, indicating that the accumulation of this important factor during defense responses is controlled by OsRAC1 via regulating both NADPH oxidase and OsCCR1 activity [18].

Another direct interactor of activated OsRAC1 is rice tryptophan-aspartic acid (WD) repeat-containing scaffold protein receptor for activated C-kinase 1, OsRACK1, a key contributor to rice innate immunity. The transcription, triggered by fungal chitin and plant hormones, and post-transcriptional events of RACK1 are positively regulated by OsRAC1, and its role in the production of ROS, most likely influenced by direct interaction with the N terminus of NADPH oxidase, has been well-documented [44]. In a two-dimensional gel electrophoresis disulfide proteomics approach, a role of OsRACK1 for being a redox status sensor in rice immunity was suggested [45]. The activity of OsRACK1 cysteine residues appears to be suppressed when OsRAC1-mediated signaling is inhibited. The contribution of OsRACK1 to resistance toward rice blast fungus is further supported by its direct interaction with distinct transcription factors. OsRap2.6 forms a complex with OsRACK1 and *Oryza sativa* mitogen-activated protein kinase 3/6 (OsMAPK3/6) in the nucleus and the cytoplasm, and an increased resistance to fungal infection suggests a positive contribution to rice immunity [46]. Nuclear-localized *Oryza sativa* CONSTANS-like 9 (OsCOL9) enhances disease resistance by regulating the expression of phytohormone signaling-related genes, especially through salicylic acid and ethylene pathways. OsCOL9 not only interacts directly with OsRACK1 in the nucleus, it also positively regulates OsRACK1 expression at the mRNA level [47]. 

In *Arabidopsis*, an ortholog of OsRACK1 has been shown to function as a scaffold to link a Gβ subunit of a heterotrimeric G-protein complex to all three tiers of an MAPK cascade, thereby facilitating immune signaling upon recognition of pathogen-secreted proteases [48]. Regarding rice, an OsRAC1-OsRACK1-mediated activation of MAPK cascades is unknown, however OsRAC1 functions immediately downstream of a heterotrimeric G-protein and OsMAPK6 protein levels were strongly reduced in an OsRAC1-silenced background [22,26], suggesting a MAP kinase cascade downstream of these two G-proteins. MAPK cascade signaling has nevertheless been shown in other OsRAC1-regulated immune reactions in rice. Rac immunity 1 (RAI1), a basic helix-loop-helix transcription factor, is involved in defense reactions against the rice blast fungus and is regulated by OsRAC1 via the OsMAPK3/6 cascade [49], suggesting a similar regulation like OsRap2.6 [46]. It could be shown that an OsRAC1-OsMAPK complex formation potentially leads to the phosphorylation of RAI1 by OsMAPK3/6 and *Oryza sativa* mitogen-activated protein kinase kinase 4 (OsMKK4), resulting in the transcriptional activation of fungal elicitor-responsive genes phenylalanine ammonia lyase 1 (*PAL1*) and *OsWRKY19* [49]. Interestingly, PAL1 expression was enhanced by overexpressing a 66-amino acid (aa) immune response peptide (IRP) in the absence of chitin as well [50]. Elevated levels of IRP also induced MAPK3/6 activation, and both biological responses were even more induced after chitin treatment. It is tempting to speculate that the IRP perception triggers an OsRAC1-regulated signaling pathway via an as-yet-unknown IRP receptor, and both signaling pathways, when triggered simultaneously, work synergistically through OsRAC1.

In a recent study, the impact of the OsRAC1-OsMAPK6 pathway on regulating grain size in rice was revealed. OsMAPK6 controls cell division and hence, grain size. The interaction with OsRAC1, however, influences the protein level and activity of OsMAPK6, leading to enhanced cell division in rice panicles [51]. Immune reactions usually consume valuable energy, resulting in a decrease of grain yield. The OsRAC1-OsMAPK6 pathway appears to achieve the delicate balancing of both worlds, disease resistance and yield, suggesting as-yet-unknown accompanying regulatory modules responsible for response fine-tuning and adaptation to any given environmental condition. Another recent study demonstrated the regulatory connection between disease resistance and plant growth [52]. The transcription of OsPT8, a Pht1 family phosphate transporter, is suppressed during MTI and infection with rice pathogens. However, OsPT8-overexpressing plants grew better under phosphate (Pi) deficiency than control plants, but MTI-responsive genes like *OsRAC1*, *PAL1* and *SGT1* were suppressed. Even though these experiments were conducted under one particular environmental condition, they already emphasized the central role that OsRAC1 plays in the network that covers diverse signaling pathways such as immunity and development. 

In ETI, RAI1 has recently been reported to be a signaling component in another NLR protein-mediated resistance pathway in rice. In this pathway, the NLR protein PID3 requires OsRAC1 to induce RAI1 expression to confer rice blast resistance [53]. Together with Pit and Pia, PID3 is yet another NLR protein that triggers ETI-mediated disease resistance in which OsRAC1 is involved. Furthermore, in a heterologous system, a dominant-negative OsRAC1 mutant was overexpressed in tobacco, which attenuated ROS production and HR development usually triggered by the resistance gene *N* or *Pto* [54]. It seems likely that the future will reveal further examples of OsRAC1 contributing to NLR-mediated disease resistance in rice. ETI, however, could also be triggered by NLR proteins that monitor the activity of the GTPase. OsRAC1 appears to be targeted by *Magnaporthe oryzae* effector protein AvrPiz-t for suppression of ROS production [55], suggesting a guardee role of OsRAC1. In any case, the question remains, how MTI and ETI, both of which appear to be fundamentally regulated by OsRAC1 in rice, can lead to completely different response amplitudes, e.g. MTI reactions often do not lead to an HR [56,57]. The answer herein might lie in the nature of the NLR proteins itself. The oligomerization of NLRs allows the formation of a funnel-structure containing a complex called the NLR resistosome that is required for plasma membrane association, cell death and eventually resistance [58]. In terms of OsRAC1-regulated ETI, it is tempting to speculate that NLR-mediated OsRAC1 activation leads to the formation of a resistosome complex in the plasma membrane by a so far undiscovered mechanism. A different hypothesis considers the regulation of OsRAC1 by Rho-GTPase activating proteins (Rho-GAPs). Previous reports about the GAP activity of SPIN6 toward OsRAC1 in rice cells indicated a U-box E3 ligase-mediated ubiquitination pathway positively regulating OsRAC1-mediated rice immune reactions [59,60]. In the absence of SPIN6, either by silencing *SPIN6* or proteasomal degradation of SPIN6 upon rice U-box E3 ligase SPL11-mediated ubiquitination, defense-related gene expression and ROS production are elevated. An increased resistance to bacterial and fungal pathogens has been observed as well, which might have been caused by the concomitant programmed cell death. This suggests that OsRAC1 activity, if uncontrolled by GAPs, could be sufficient for triggering programmed cell death, and NLR activity supporting proteasomal SPIN degradation might be one mechanism to achieve elevated OsRAC1-mediated signaling.

## 3. Barley *HvRACB* Acts in Cell Polarity and Susceptibility to Powdery Mildew

The function of the metazoan Rho family protein RAC1 in regulating the respiratory burst NADPH oxidase complex motivated research on RAC1-like ROPs in barley (*Hordeum vulgare*), because reactive oxygen species are involved in epidermal cell penetration success or failure of the biotrophic barley powdery mildew fungal parasite *Blumeria graminis* f.sp. *hordei*. Both single cell silencing of the barley ROP *HvRACB* and overexpression of a constitutively activated HvRACB mutant supported that HvRACB acts in susceptibility to fungal penetration [61,62]. The susceptible host appears to promote fungal accommodation and forms a new apoplastic compartment around the fungal feeding organ, the haustorium. This compartment possesses a cell wall-like extrahaustorial matrix and an extrahaustorial host membrane in continuum with the host plant plasma membrane. The ingrowth of the haustorium into epidermal host cells has been compared to the outgrowth of root hair and pollen tubes, which also requires coordinated function of plant HvRACB-like ROPs of type I [63,64]. In these developmental programs, ROPs organize the filamentous F-actin and microtubule cytoskeleton as well as vesicle trafficking and fusion at the growing cell tip [65,66]. Indeed, stable silencing of *HvRACB* in transgenic barley plants strongly suggested that the physiological function of HvRACB is involved in developmental processes rather than in controlling host immune signaling. This was concluded because *HvRACB*-silenced plants allow for less and smaller haustoria and are unable to form root hairs by trichoblast outgrowth. In addition, these plants show failure in asymmetric cell division in the leaf epidermis, which likewise requires ROPs in other grass species [11,67,68]. The *HvRACB* silenced plants, however, show wild-type-like activation of early pattern-triggered immune responses to fungal chitin and the bacterial flagellin peptide flg22. Along that line, transgenic barley expressing constitutively activated HvRACB shows wild-type-like pattern-triggered immune responses and largely unaltered cell wall-associated H_2_O_2_ production at sites of fungal attack, but shortened polar growth of root hairs. By contrast, constitutively activated HvRAC1 appears to have the potential to support cell wall-associated defense and the hypersensitive cell death reaction when overexpressed in barley. Astonishingly, this potential of HvRAC1 is not associated with enhanced resistance to *B. graminis* f.sp. *hordei*, but to *M. oryzae* [68,69]. Additionally, misexpression of *HvRACB* or HvRACB-binding HvMAGAP1 (microtubule-associated ROP-GAP1) influenced the ability of barley epidermal cells to polarize their F-actin or microtubule cytoskeleton to sites of fungal penetration attempts [11,64]. Together, the data strongly support that barley HvRACB is a key factor in epidermal cell polarity of barley, and this function may also support cellular accommodation of fungal infection structures. Indeed, *B. graminis* f.sp. *hordei* expresses an unconventional virulence effector protein, ROPIP1 (ROP-interactive peptide 1), that can enter barley epidermal cells via an unknown mechanism and interact with HvRACB. The ectopic expression of ROPIP1 in barley destabilizes the microtubule cytoskeleton and supports fungal cell entry. Therefore, HvRACB is considered to be a fungal virulence target in effector-triggered susceptibility [70].

### 3.1. Activation and Inactivation of HvRACB 

**Figure 3:**
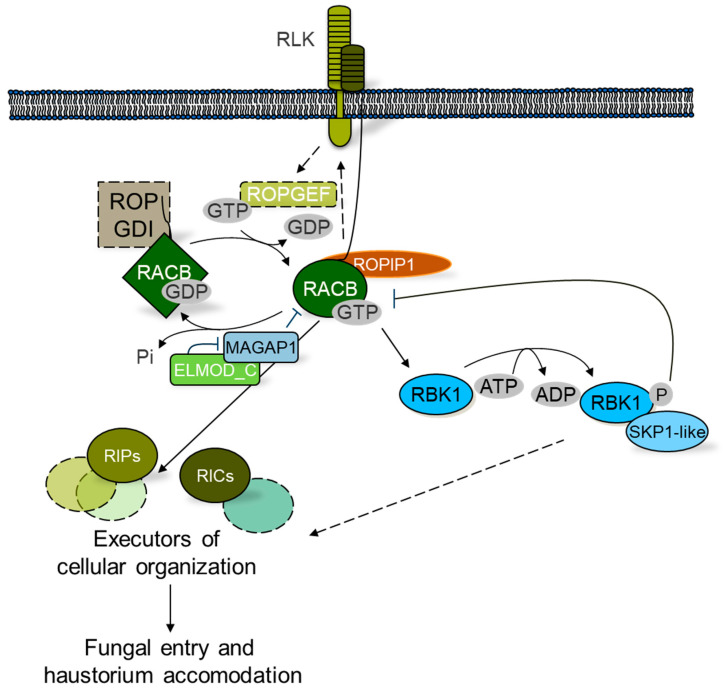
Barley cell surface ROP signaling in susceptibility to parasitic fungal cell entry. The barley ROP protein HvRACB might be activated from the cell surface and supports fungal entry into barley epidermal cells with support from RIC and RIP scaffold proteins. The fungal effector ROPIP1 can directly bind RACB and support host cell entry. RACB activity and abundance are further controlled via MAGAP1 and RBK1, respectively. Postulated components are bordered with dashed lines. Dashed arrows show indirect, postulated or speculative interactions.

Constitutively activated, but not dominant-negative, HvRACB supports fungal penetration success [63]. This supports that HvRACB has to be activated to serve fungal demands. However, it is not known how HvRACB is activated in the authentic interaction with *B. graminis* f.sp. *hordei*. Several scenarios appear to be possible. Fungal effectors such as ROPIP1 may play a role in activating the G-protein or in arresting RACB in its GTP-bound activated form [70]. Alternatively, HvRACB is activated via endogenous ROP signaling involving GEFs. This appears to be plausible because HvRACB-like type I ROPs are activated via PRONE GEFs in pollen tube growth, root hair growth and cell wall integrity signaling. In these processes, RLKs such as pollen receptor kinase 2 or FERONIA activate GEFs via phosphorylation, which releases autoinhibition of the GEF’s PRONE domain [71,72]. The RLK FERONIA is also a cell wall integrity sensor [73] and a susceptibility factor of *Arabidopsis* to powdery mildew [74], but its function in grass powdery mildew disease is unknown. Presumably, cell wall sensing is switched on during fungal cell wall penetration and many RLKs are expressed in barley interaction with *B. graminis* f.sp. *hordei.* Additionally, RLKs are overrepresented in the set of genes which are HvRACB-dependently expressed at the transcript level, and some of these RLKs also support fungal cell entry. It was therefore suggested that activated HvRACB supports the expression of RLKs that can again activate HvRACB via GEFs in a positive feedback loop that is of use for the fungus [75] (Figure 3).

Barley HvMAGAP1 was found in yeast two-hybrid screening using HvRAC1 or HvRACB as bait proteins [11]. HvMAGAP1 contains a conserved CRIB (Cdc42/Rac-interactive binding) motif for binding GTP-bound ROPs and a conserved GAP domain for activation of ROP GTPase function. The GAP domain contains a conserved arginine residue that is predicted to reach as a finger-like protrusion into the GTP-binding pocket of the GTPase to facilitate GTP hydrolysis. HvMAGAP1 is further associated with microtubules via its C-terminus, a feature that is not conserved in dicotyledonous plants. Interestingly, HvMAGAP1 supports resistance to fungal entry and polar organization of the microtubule cytoskeleton, but only if the catalytic arginine finger of HvMAGAP1 is intact. Together, switching off HvRACB by GTP hydrolysis apparently functions in penetration resistance [11].

ROP signaling activity can be regulated beyond GTP/GTP loading/hydrolysis by post-translational modifications. These modifications can give rise to localization in membrane domains (lipidations), protein–protein interactions including those with GEFs (phosphorylation, see also 3.2.) [76], or protein stability (ubiquitination, see also 3.2.). HvRACB is possibly both constitutively prenylated at its C-terminus and reversibly S-acylated at a conserved cysteine residue [9,77]. HvRACB´s C-terminal CAAX box (cysteine, aliphatic, aliphatic, any residue) prenylation motif CSIL appears functional because the truncation of those four amino acids renders the protein non-functional as a susceptibility factor and leads to cytoplasmic, instead of plasma membrane, localization of the protein [63,77].

### 3.2. Scaffolds and Executors of HvRACB Function in Susceptibility or Resistance

Activated GTP-loaded ROPs signal downstream via effectors or executors (we prefer the term executors here to avoid confusion with pathogen virulence effectors). Additionally, activated ROPs may assemble executor complexes with the support of scaffold proteins that form molecular clamps bringing the G-proteins and downstream factors together. In barley, several proteins were shown to interact preferentially with the activated form of HvRACB and hence are considered to be potential downstream factors (Figure 3). The receptor-like cytoplasmic kinase of class RLCK VI_A, HvRBK1 (ROP binding protein kinase 1), was identified in yeast two-hybrid screening with HvRACB as a bait protein. HvRBK1 was then shown to interact with constitutively activated HvRACB in planta and to be recruited by constitutively activated HvRACB to the plasma membrane. The in vitro kinase activity of HvRBK1 is low, but can be boosted by addition of activated HvRACB. Hence, HvRBK1 is a ROP-binding and ROP-activated kinase. However, silencing experiments suggest that HvRBK1 acts in stabilizing microtubules and in resistance to fungal penetration [78]. This could be explained by a hidden function of HvRACB signaling in resistance or by a negative regulatory function of HvRBK1 in HvRACB signaling. The latter is supported because HvRBK1 interacts with HvSKP1-like protein, a predicted subunit of an SCF E3 ligase complex that mediates protein ubiquitination. Silencing of either *HvRBK1* or *HvSKP1-like* enhanced protein abundance of activated HvRACB in barley epidermal cells along with enhanced susceptibility [79]. Hence, there might be a negative feedback of ROP-activated HvRBK1 on protein abundance of HvRACB. Interestingly, phosphorylation and SCF-mediated ubiquitination also regulate mammalian RAC1 signaling [80]. Most recently, in vitro phosphorylation of HvRACB by HvRBK1 was shown. Additionally, the conserved mammalian RAC1 ubiquitination site is also ubiquitinated in plant HvRACB as evidenced by mass spectrometry. However, it remains unclear whether HvRBK1 phosphorylates HvRACB *in vivo* and whether this makes HvRACB a substrate for ubiquitination [77]. It is noteworthy that HvRAC1 can also interact with HvRBK1 and activate the kinase in vitro [78]. This raises further the possibility that HvRBK1 function in resistance can be explained by a function downstream of HvRAC1.

RICs are ROP-interactive CRIB motif-containing proteins that act as ROP scaffolds [81]. Barley encodes at least eight RIC proteins with a conserved CRIB motif, but little sequence similarities outside that ROP-binding motif. Barley HvRIC171 (named after the number of predicted amino acid residues encoded by the coding sequence) interacts with HvRACB in yeast and in planta and induces super-susceptibility to fungal penetration when overexpressed. Both proteins might act in a similar fashion or in the same signaling pathway because susceptibility cannot be further enhanced by co-expression of constitutively activated HvRACB [82]. Similarly, HvRIC157 preferentially interacts with activated HvRACB in planta and enhances susceptibility to fungal penetration in an HvRACB-dependent manner. HvRACB and HvRIC157 together localize at the cell periphery or plasma membrane where *B. graminis* f.sp. *hordei* invades susceptible barley cells [83]. Similarly, HvRACB and HvRIC171 form a protein complex at sites of fungal entry [84]. Although no RIC interactors besides ROPs have been identified in barley so far, data suggest that HvRACB executor complexes form at specific subcellular locations, which may be triggered during fungal attack. Future research can now make use of RICs that function in susceptibility to find additional components of HvRACB executor complexes.

Another class of CRIB motif-containing proteins in plants is a subfamily of ROP-GAPs [85], of which HvMAGAP1 is a prime example that, as mentioned before, limits susceptibility to *B. graminis* f.sp. *hordei.* However, it also acts in symmetry breaking of plasma membrane domains and microtubule organization together with HvRAC1 and HvRIPa (an ICR/RIP scaffold, interactor of constitutive active ROPs, synonym: ROP-interactive partner). In this function, HvMAGAP1 combines ROP-GAP and ROP executor functions because microtubule organization is greatly influenced by HvMAGAP1 [86]. This opens a new perspective on plant CRIB-GAPs as both regulators and downstream factors of activated ROPs. HvMAGAP1 itself appears also to be regulated by its protein interactor HvELMOD_C, a plant Engulfment and Motility domain-containing protein, that co-localizes with HvMAGAP1 at microtubules, when both proteins are co-expressed. In this experimental setup, HvELMOD_C also counteracts the effect of HvMAGAP1 in penetration resistance [87]. In animals and slime molds, ELMO domain proteins are key factors in the activation of Rho signaling [88,89]. 

ICR/RIP proteins are another class of ROP scaffolds [10]. Barley encodes three ICR/RIPs, of which only HvRIPb appears to function in susceptibility to *B. graminis* f.sp. *hordei* [90]. HvRIPb can homodimerize and bind to both HvRACB and microtubules via separated coiled-coil protein domains. The HvRACB-binding domain alone is associated with HvRACB at the plasma membrane and supports fungal penetration when overexpressed as a truncated HvRIPb protein variant. Similar to what is observed for specific barley RICs, HvRIPb and its HvRACB-binding domain localize to sites of fungal penetration. All of these proteins preferably or exclusively interact with activated HvRACB, which suggests high activity of HvRACB or related ROPs at fungal entry sites. This brings us back to the question of whether intrinsic ROP signaling is activated at these sites or the fungus interferes with host ROP signaling by virulence effector-mediated activation of ROPs. Together, HvRACB may be the core of a signaling interface that is co-opted for fungal accommodation in intact barley cells. More detailed understanding of HvRACB upstream and downstream signaling will allow for a deeper insight into how grass ROPs operate and how pathogens manipulate plant ROPs. This could also bear hidden functions of HvRACB signaling in pathogen resistance, e.g. via HvRACB´s function in cytoskeleton organization which is also involved in cell wall-associated defense [91,92], or more mechanisms that control HvRACB during successful fungal infection. Eventually, we may understand why *B. graminis* f.sp. *hordei* can profit from a host ROP GTPase and whether this indeed resembles a kind of hostile takeover of a plant cell developmental program. 

## 4. An Excursion into Mammalian Rho Signaling in Bacterial Pathogen Entry and Immunity

The pivotal role that Rho GTPases play during certain interactions with microbes is not restricted to plants alone. Despite important differences in the pathogenesis of plants and animals, which are caused, among others, by the presences of a rigid plant cell wall as a physical barrier for pathogen invasion and the fact that plant-pathogenic bacteria do not enter intact host cells, there are conceptual and mechanistic similarities between the roles of plant and animal Rho family proteins in disease and immunity. In fact, specifically for infectious diseases of mammals, Rho GTPases have been extensively documented to be exploited to promote either invasive or antiphagocytic activities of bacterial pathogens [93,94]. Both activities require the manipulation of host cell processes that govern cytoskeleton rearrangements. During infection, via the type III secretion system, Gram-negative bacteria inject effector proteins into the host cytosol to target host proteins in order to promote invasion and to create a replicative environment [95]. Since Rho proteins are signaling hubs that can steer cytoskeleton dynamics in animals as well, Rho-dependent pathways are therefore highly attractive targets for bacterial effector proteins. The resulting effector-triggered susceptibility via targeting Rho proteins has been shown for quite a few bacterial effector proteins. The internalization of the enteropathogenic bacterial pathogen *Salmonella enterica* serovar Typhimurium (*S*. Typhimurium) requires actin remodeling that is achieved upon translocation of several type III effector proteins. While SipA and SipC prevent actin depolymerization by directly binding to actin filaments [96], the effector proteins SopE/E2 modulate actin organization indirectly by binding to Rho GTPases which regulate actin polymerization via the Arp2/3 complex [97,98]. SopE/E2, thereby, benefit from an intrinsic guanine nucleotide exchange factor activity which leads to the activation of the host Rho GTPases Cdc42 and Rac1 [97,99,100,101]. SopB, another effector protein of *S*. Typhimurium, aids in the infection of epithelial cells by activating the Src homology 3 domain (SH3) GEF, which in turn activates RhoG that is involved in actin remodeling [98]. Furthermore, the lipid phosphatase activity of SopB results in the recruitment of RhoB, RhoH and RhoJ to bacterial invasion sites, where the activity of these GTPases have been shown to contribute to bacterial invasion [102]. For *Yersinia* bacteria, in the antiphagocytic part of the life cycle, the inhibition of phagocytosis is achieved by targeting the same Rho GTPases involved in actin modeling which are required for bacterial invasion [94]. The type III secreted effector protein YopE was shown to harbor a strong GAP activity toward Rac1 and RhoG [103,104], while the cysteine protease activity of YopT leads to the removal of Rho proteins from the plasma membrane upon proteolytical cleavage of the lipid-modified C-terminal cysteine [105], indicating a tightly controlled activity of the effector protein repertoire in both invasive and antiphagocytic life phases of the bacteria.

Host cells, however, have the ability to monitor changes in Rho activity, thereby indirectly recognizing an intruder. The molecular mechanisms behind the induction of inflammatory responses are reminiscent of ETI in plants, where NLRs detect changes in the metabolic state of a guarded protein, the guardee [13,106]. Cytotoxic necrotizing factor 1 (CNF1) from *Escherichia coli*, although not a classical type III secreted effector, destroys the GTPase activity of Rac2, RhoA and Cdc42 by deamidating certain glutamine residues, thereby rendering these GTPases permanently activated. CNF1 presence, however, also induces inflammatory reactions like nuclear factor-κB (NF-κB) activation and cytokine secretion, through the innate immune adaptors, IMD in *Drosophila* and RIP2 in mammals [107,108]. Similar inflammatory reactions are also triggered by the abovementioned *Salmonella* effector protein SopE [109,110]. Crucial for this immune response is the activation of nucleotide-binding oligomerization domain NLRs, NOD1 and NOD2, that have long been known to recognize certain structures within bacterial peptidoglycan [111]. Interestingly, there is accumulating evidence that NF-κB activation and hence an inflammatory response via NOD1 and NOD2 can occur independently of peptidoglycan [112]. In fact, NOD1 and NOD2 are assumed to be activated by the disruption of the actin cytoskeleton and the activity of Rac1 and Cdc42 and other Rhos as well, suggesting a guardee role of these GTPases [110,113]. A direct interaction between Rac1 and NOD1 in human cells was demonstrated, which is along the lines of the OsRac1-Pit interaction in rice, emphasizing a comparable interkingdom evolutionary background of immunity based on Rho GTPase activity sensing by NLRs [32,114]. Together, in both the plant and animal kingdoms, host Rho GTPases are targeted by pathogen virulence effectors for aberrant regulation of cellular functions and they share functions in ETI as they operate as guardees or downstream signaling proteins of NLR immune receptors.

## 5. ROPs Involved in Further Plant-Microbe Interactions

In contrast to HvRACB and OsRAC1, the knowledge of ROPs from other plants that regulate either positively or negatively the cellular responses to invading microbes is less comprehensive but promising for future discoveries. Research especially in *Arabidopsis* concentrated on ROP-mediated developmental signaling, in particular the control of cytoskeleton activities leading to cell polarization events that are required for root-hair development and pollen tube growth [115]. However, several ROPs in different plants have highlighted their importance not only in pathogenesis, but also symbiosis, and below we aim to summarize the current information.

### 5.1. The Role of Arabidopsis ROP6 in Response to Powdery Mildew

Eleven ROP family members have been described, of which AtROP2, AtROP4 and AtROP6 regulate auxin-dependent polar cell growth [115,116]. AtROP6, in particular, is involved in the organization of cortical microtubules through activation of katanin via RIC1 and also in F-actin bundle formation [117,118]. SPIKE1, a DOCK family GEF, was shown to act upstream of AtROP6 and is most likely responsible for its activation [119,120]. The AtROP6 activity might be further regulated by physically interacting with phosphatidylglycerol, that has been shown to inhibit AtROP6-controlled endocytotic processes [121]. The involvement of AtROP6 in pathogenesis was nicely shown with experiments using plants expressing a dominant-negative form of this small GTPase. Those plants were less susceptible to virulent powdery mildew *Golovinomyces orontii*, but more susceptible to non-adapted *B. graminis* f.sp. *hordei* and overexpressed several defense genes. Genetic interaction studies indicated that the observed defense responses to both host and non-host powdery mildew pathogens are independent of defense signaling mediated by salicylic acid [122]. It is currently unknown which signaling cascade AtROP6 is regulating cellular responses during pathogenesis. However, both AtROPGAP1 and AtROPGAP4 can interact with AtROP6 and limit susceptibility to powdery mildew [11,123], indicating a potential negative regulatory role for AtROP6 activity. AtRLCK VI_A3, a receptor-like cytoplasmic kinase similar to barley HvRBK1, has been shown to interact directly with AtROP6 in yeast and also shows an increased in vitro kinase activity in the presence of the activated form of AtROP6. Moreover, *Arabidopsis* plants lacking AtRLCK VI_A3 are characterized by a slightly higher susceptibility toward infection by the virulent powdery mildew fungus *Erisyphe cruciferarum*, indicating that either AtROP6 might signal through AtRLCK VI_A3 during pathogen response or AtRLCK VI_A3 has a HvRBK1-like function in regulating AtROPs [124]. Most recently, AtRLCK VI_A3 and AtRBK1 have been shown to function in casparian strip organization and shaping the root-associated microbiome [125], which indicates the general complexity of ROP-regulated plant–microbe interactions. Similar to OsRAC1 and HvRACB, AtROP6 might be involved in MTI by directly interacting with *Arabidopsis* respiratory burst oxidase homolog D (AtRBOHD). *Arabidopsis rop6* mutants showed reduced levels of ROS compared to wild-type plants, while ROS levels were much higher in AtROP6-overepressing plants [126], suggesting AtROP6-mediated ROS signaling via AtRBOHD.

### 5.2. ROP GTPases Involved in Symbiosis

Legume root nodule formation is crucial for establishing an intracellular symbiotic interaction with nitrogen-fixing soil bacteria. It also shows similarities on the small G-protein signaling level to the invasion of enteropathogenic bacteria into mammalian cells. Upon chemical crosstalk of plant-derived flavonoid compounds and bacterial lipochito-oligosaccharide Nod (nodulation) factors, legume root hairs undergo morphological changes to form infection threads and eventually, mature nodules [127]. Specific Nod factor receptor kinases perceive the Nod factors in the rhizosphere, NFR1 and NFR5 in *Lotus japonicus* and LYK3, LYR3 and NFP in *Medicago truncatula* [128,129,130]. Interestingly, these symbiosis-relevant legume receptors share the highly conserved lysin motif (LysM) domain with the chitin receptors OsCEBiP/OsCERK1 that are required for fungal pathogen recognition [28,131]. The root hair deformation during nodule development is indicative of a highly controlled polarized cell growth, and the discovery of small GTPases participating in its regulation was anticipated. ROP6 from *L. japonicus* (LjROP6) which is homologous to AtROP6, has been shown to interact with NFR5, but not NFR1, in *vitro* and in planta in a GTP-binding dependent way [132,133]. The positive regulatory role of LjROP6 during the symbiotic relationship with *Mesorhizobium loti* was confirmed by root hairs either overexpressing a dominant-negative mutant form or expressing *ROP6* RNA interference constructs, both of which lead to a decrease in nodule numbers per root. In a similar approach, overexpressing a constitutively active LjROP6 resulted in a higher nodule number due to an increase in root hair curling. The mechanism of LjROP6-regulated nodule formation is still not fully characterized, but the coating protein clathrin appears to play an important role in legume symbiosis with rhizobia. Clathrin heavy chain 1 interacts directly with LjROP6, indicating that clathrin-mediated endocytosis is involved in nodulation [134]. This was further supported by studies using inhibitors of clathrin-mediated endocytosis, which gave rise to suppression of early nodulation gene expression combined with a reduction of *M. loti* infection.

In *Medicago truncatula*, the expression analysis in roots upon infection with rhizobia revealed that at least some ROP GTPases are involved in the regulation of the early rhizobial infection stage [135]. Even though the expression level of *MtROP9* does not significantly change during rhizobial infection, studies using *M. truncatula* roots transiently expressing an *MtROP9* RNA interference construct (MtROP9i) revealed that the absence of MtROP9 negatively affects rhizobial infection, while early mycorrhizal and oomycete root colonization is promoted [136]. Additionally, MtROP9i transgenic lines were impaired in the generation of ROS, probably due to the suppression of *MtRBOH* gene expression. This was further supported by a proteomics approach, which revealed a significant reduction of defense-related proteins linked to ROS production in MtROP9i [137]. MtROP10, which is transiently induced by rhizobial infection, appears to play a major role in polarized root hair growth and deformation, not only due to its subcellular localization in the absence and presence of NFs [135,138]. MtROP10 interacts with NF receptors and the overexpression of a constitutively active form promotes depolarized root hair growth. In contrast to *MtROP10*, *MtROP8* is downregulated during the early stages of rhizobial infection, but significantly upregulated in nodules [135]. RNAi-mediated *MtROP8* silencing resulted in abnormal root morphology due to changes in ROS distribution. Interestingly, the absence of MtROP8 supported infection by *Sinorhizobium meliloti* and subsequent nodulation [139]. So far, it is still unclear how activation is achieved upon interaction of MtROPs with NF receptors. However, MtRopGEF2 has been reported to interact with a couple of MtROPs [140], suggesting a receptor-controlled GEF pathway. The different MtROP expression signatures during rhizobial infection argue for a temporally and spatially controlled regulation of nodule formation. Additionally, the simultaneous involvement of ROPs in regulating ROS production points toward certain functions in MTI responses and the presence of several ROPs in legume roots is probably not a result of redundancy, but rather indicates the delicate fine-tuning of the plant coping with friends and foes at the same time.

## 6. Concluding Remarks

Research on plant Rho GTPases is still in its early days, but has made enormous progress in recent years. This is particularly true for elucidating the role of ROPs in plant development, but ROP functions in response to biotic as well as abiotic environments are increasingly recognized. The complexity of Rho signaling in animals appears to find its renaissance in plants, and plant-specific components are adding another perspective on general conserved, kingdom- and even plant lineage-specific Rho signaling. Rho functions seem to be cross-kingdom comparable in support and defense of microbial pathogens. However, the molecular details of these functions may highly depend on the individual pathosystem and the pathogen´s specific effector repertoire. We therefore look forward to the future discovery of more Rho functions in plant disease and immunity and foresee that pathogens and the beneficial microbiome will teach us a lot more about how Rho proteins function.
